# RETRACTED: Sarnella et al. A Novel Inhibitor of Carbonic Anhydrases Prevents Hypoxia-Induced TNBC Cell Plasticity. *Int. J. Mol. Sci.* 2020, *21*, 8405

**DOI:** 10.3390/ijms262311388

**Published:** 2025-11-25

**Authors:** Annachiara Sarnella, Giuliana D’Avino, Billy Samuel Hill, Vincenzo Alterio, Jean-Yves Winum, Claudiu T. Supuran, Giuseppina De Simone, Antonella Zannetti

**Affiliations:** 1 CNR Istituto di Biostrutture e Bioimmagini, 80122 Napoli, Italy; 2 IBMM, Universite Montpellier, CNRS, ENSCM, 34296 Montpellier, France; 3 Dipartimento NEUROFARBA, Sezione di Scienze Farmaceutiche, Università di Firenze, Sesto Fiorentino, 50139 Firenze, Italy

The journal retracts the article “A Novel Inhibitor of Carbonic Anhydrases Prevents Hypoxia-Induced TNBC Cell Plasticity” [1] cited above.

Following publication, concerns were brought to the attention of the Editorial Office regarding the integrity of a number of images presented in this publication [1].

Adhering to our standard procedure, an investigation was conducted by the Editorial Office and Editorial Board that confirmed partial duplication in Figure 7 as well as partial duplication between Figure 6 presented in this article [1] and an earlier publication [2], representing different experimental conditions. Consequently, the Editorial Board has lost confidence in the reliability of the findings and has decided to retract this publication, as per MDPI’s retraction policy (https://www.mdpi.com/ethics#_bookmark30 (accessed on 29 October 2025)).

This retraction was approved by the Section Editor-in-Chief of *International Journal of Molecular Sciences.*

The authors did not agree to this retraction.
